# Effectiveness of Epidermal Growth Factor Loaded Carboxymethylcellulose (EGF-CMC) Hydrogel in Biofilm Formation in Wounds of Diabetic Patients: A Randomized Clinical Trial

**DOI:** 10.3390/gels9020117

**Published:** 2023-02-01

**Authors:** Fernanda Soares Pessanha, Beatriz Guitton Renaud Baptista de Oliveira, Bianca Campos Oliveira, Gabriela Deutsch, Felipe Lopes Teixeira, Luciana Castilho Bokehi, Mariana Alcântara Calomino, Selma Rodrigues de Castilho, Rossana Mara da Silva Moreira Thiré, Lenise Arneiro Teixeira, Geraldo Renato de Paula

**Affiliations:** 1 Nursing School, Universidade do Estado do Rio de Janeiro, Rio de Janeiro 20551-030, Brazil; 2 Aurora de Afonso Costa College of Nursing, Universidade Federal Fluminense, Niterói 24020-091, Brazil; 3 College of Pharmacy, Universidade Federal Fluminense, Niterói 24241-000, Brazil; 4 COPPE/Program of Metallurgical and Materials Engineering—PEMM, Universidade Federal do Rio de Janeiro, Rio de Janeiro 21941-599, Brazil

**Keywords:** epidermal growth factor, biofilm, *Staphylococcus aureus*, *Pseudomonas aeruginosa*, diabetic foot

## Abstract

Diabetic patients frequently develop wounds, which can be colonized by bacteria, mainly *Staphylococcus aureus* and *Pseudomonas aeruginosa*, with the ability to form biofilms. This study aimed to evaluate the colonization and biofilm formation of *Staphylococcus aureus* and *Pseudomonas aeruginosa* in chronic wounds of diabetic patients treated with a bioactive dressing (EGF-CMC), which consisted of a 2% carboxymethylcellulose (CMC) hydrogel loaded with epidermal growth factor (EGF). This randomized clinical trial was conducted with 25 participants: 14 treated with EGF-CMC hydrogel and 11 treated with CMC hydrogel for 12 weeks. Participants with type 2 diabetes mellitus were selected. All had diabetic foot ulcers or chronic venous ulcers. Swab collections were performed on weeks 1, 6, and 12. The laboratory analyses included the identification of strains, microbial quantification, virulence gene investigation, and the evaluation of biofilm formation. In total, 13 *S. aureus* strains and 15 *P. aeruginosa* strains were isolated. There were no statistically significant differences regarding bacterial loads and virulence genes. However, EGF-CMC-hydrogel-treated wounds were colonized by strains with lower biofilm formation abilities. The probability of isolating biofilm-producing strains from CMC-hydrogel-treated wounds was 83% greater than the probability of isolating biofilm-producing strains from EGF-CMC-treated wounds.

## 1. Introduction

The prevalence of chronic wounds is estimated at 1.67 per 1000 inhabitants of the general population, largely composed of diabetes mellitus patients [1]. The estimated cost of wound care for diabetic patients reaches up to USD thirteen billion annually in the United States [2]. The number of diabetic patients is growing [3], and it is known that changes in skin integrity can be prevalent in up to 34% of cases [4]. Hyperglycemia-induced microvascular dysfunction may be one of the major causes of diabetic complications [5], which reiterates the importance of a treatment program with standardized therapeutic components, including metabolic control, the debridement of necrotic tissues, and the application of dressings [4].

Modern dressings, such as hydrogels, are biocompatible, degradable, and present moisture retention ability in clinical practice. Thus, they can provide a physical barrier to protect the wound and a moist environment for wound healing, promote pain relief, and improve the hypoxic or anaerobic environment in the wound bed [6].

Hydrogels are three-dimensional networks that can swell in water or biological fluids and can hold large amounts of liquids. They are hydrophilic polymers formed through physical or chemical crosslinks [7,8]. Thus, the main components of this solid-like structure are an elastic crosslinked network and a solvent. In an aqueous medium, swollen hydrogels mimic the extracellular matrix (ECM) of living tissues [6]. These properties make hydrogel-based materials good candidates for wound dressing applications. Several synthetic or bio-based matrices can be used in hydrogel dressings, including carboxymethylcellulose (CMC).

In particular, 2% carboxymethylcellulose hydrogel is considered a standard treatment for chronic wounds in diabetic patients [9]. Carboxymethylcellulose (CMC) is a polysaccharide derived from the chemical modification of cellulose. Its chemical structure is composed of carboxymethyl groups (-CH2-COOH) bound to the hydroxyl groups of the glucopyranose chain of cellulose. It is non-toxic for humans, abundant in nature, and cost-effective. CMC presents strong hydrophilicity due to its carboxyl and hydroxyl groups and high absorption capacity, which helps the proliferation and migration of fibroblasts and keratinocytes, provides the autolytic debridement of necrotic tissue and the removal of foreign bodies, and impairs bacterial growth [10].

In recent years, a better understanding of the biological steps in the wound healing process motivated the development of the next generation of biologically enhanced wound dressings. These bioactive dressings play an active role in the healing process by activating or driving the appropriate physiological responses required for cellular regeneration and tissue reconstruction in wounds. This biological response can be achieved by incorporating delivery systems of active agents, such as antimicrobial agents, growth factors, and cells [10].

Thus, these dressings can combine the protection and moisture control of modern dressings with the abilities of specialized bioactive molecules to stimulate cell regeneration, increase collagen synthesis, combat bacterial infections, and provide drug delivery functions for enhancing the wound healing process. For example, a prior study on an in situ injectable hydrogel loaded with quaternary ammonium and fibroblast growth factor (FGF) found good in vivo and in vitro efficacy concerning antimicrobial activity [11].

Other possibilities include improving the therapeutic effects of hydrogel-based dressings with molecules such as epidermal growth factor (EGF), vascular endothelial growth factor (VEGF), and platelet-derived growth factor (PDGF) [8]. These bioactive molecules play direct roles in wound closure, epithelization, ECM deposition, and neovascularization processes.

Epidermal growth factor (EGF) is one of the bioactive molecules associated with wound dressings. EGF interacts with a receptor tyrosine kinase, activating a signaling cascade that results in successive biochemical changes that lead to the proliferation of keratinocytes, the stimulation of angiogenesis, and the activation of fibroblasts [12], which are essential for wound healing. Previous studies have demonstrated the good tolerability and safety of continuous EGF injections in humans and experimental animals [13,14].

The choice of appropriate delivery systems is a key aspect regarding the use of growth factors in wounds, as these molecules need to remain bioactive to achieve wound healing. EGF tends to degrade when exposed to proteinase and oxygen in the wound environment [15]. A prior study showed that incorporating EGF into a CMC hydrogel effectively enhanced chronic wound healing, especially the healing of diabetic ulcers, reducing the wound area and improving the tissue and exudate quality [16].

Diabetic patients frequently develop wounds, which can be colonized by bacteria, mainly *Staphylococcus aureus* and *Pseudomonas aeruginosa* [17,18]. These bacteria have numerous virulence factors, including the ability to form biofilms [19,20,21]. Biofilms are structures composed of aggregated microorganisms attached to wound surfaces that accumulate a protective extracellular polysaccharide matrix (EPS) to optimize, for example, the use of available nutritional resources. The microorganisms of biofilms have increased antibiotic tolerance and evaded host immune defenses [17]. Thus, the presence of biofilms is one of the major contributors to impaired wound healing.

Microbiological evaluations of wounds treated with growth factors were performed in preclinical studies [11,22]. A 2019 study addressed wound care with EGF, considering the outcomes of reduced colonization and infection by *S. aureus* and *P. aeruginosa*. The authors found a higher prevalence of the isolation of *S. aureus* strains at the beginning of follow-up, while there was an increase in the isolation of *P. aeruginosa* strains at the end of treatment with EGF [23]. This reiterates the novelty of studies on this topic.

In this context, this study aimed to evaluate the colonization and biofilm formation of *Staphylococcus aureus* and *Pseudomonas aeruginosa* in chronic wounds of diabetic patients treated with a bioactive dressing (EGF-CMC) that consisted of a 2% carboxymethylcellulose (CMC) hydrogel loaded with epidermal growth factor (EGF).

## 2. Results

In total, 25 patients were followed in the study: 14 in the EGF group (56%) and 11 in the hydrogel group (44%). The sample was evenly distributed concerning the treatment (binomial test, *p*-value = 0.690).

### 2.1. Health History and Wound Characteristics

Participants with type 2 diabetes mellitus were selected. They were male, aged between 52 and 70 years, and had uncontrolled glycated hemoglobin (greater than 7%) and a normal ankle–arm index (ABI) (greater than 0.91). Their lesions had partial depth (100%, 25/25) and showed no edema (92%, 23/25), pruritus (96%, 24/25), necrosis (100%, 25/25), heat (100%, 25/25), or odor (100%, 25/25). Table 1 shows the characteristics of the wounds and the health history of the patients, showing that the groups were homogeneous.

The median conditions of the patients were 64 years old (interquartile range (IR) = (54.0; 69.0)); injury area equal to 7 cm^2^ (IR = (4.0; 27.6)); granulation equal to 62.3% (IR = (43.7%; 78.7%)); and slough equal to 13.5% (IR = (0.0%; 36.9%)).

### 2.2. Identification of S. aureus and P. aeruginosa Strains

Table 2 shows the incidence of colonization by *S. aureus* and *P. aeruginosa* in the EGF-CMC and CMC groups. Eight isolates of *S. aureus* were identified in the CMC group, while five isolates were obtained in the EGF-CMC group. The mean identification of *S. aureus* was 0.36 per patient in the EGF-CMC group and 0.73 per patient in the CMC group. There was no significant difference between the groups (*p*-value = 0.241).

Regarding the incidence of colonization by *P. aeruginosa*, 11 isolates were identified in the EGF-CMC group, and 4 were identified in the CMC group. The mean identification of *P. aeruginosa* was 0.79 per patient in the EGF-CMC group and 0.36 per patient in the CMC group. There was no significant difference between the groups (*p*-value = 0.227 using Fisher’s exact test). In 7 of 25 patients, neither *S. aureus* nor *P. aeruginosa* were isolated.

### 2.3. Antimicrobial Susceptibility

Regarding the analysis of the resistance of *P. aeruginosa* strains to antibiotics, no strain isolated from the CMC group (0.0%) showed resistance to any antibiotic; all were sensitive to all tested antibiotics. On the other hand, two strains isolated from wounds treated with EGF-CMC hydrogel (18.2%) showed resistance to at least one antibiotic. One strain was resistant to aztreonam and ciprofloxacin, and the other strain was considered multidrug-resistant, as it showed resistance to all tested antimicrobials (aztreonam, ciprofloxacin, levofloxacin, gentamicin, meropenem, imipenem, ceftazidime, and piperacillin with tazobactam) except for polymyxin B. There was no significant difference in the resistance frequencies between treatment groups (*p* value = 1.000 using Fisher’s exact test).

Regarding the *S. aureus* samples, seven strains from the CMC group (87.5%) were resistant to at least one antibiotic: 25% (2/8) were resistant to chloramphenicol, 25% (2/8) were resistant to cefoxitin, 75% (6/8) were resistant to ciprofloxacin, and 100% (8/8) were resistant to penicillin. Three strains from the EGF-CMC group (60.0%) were resistant to at least one antibiotic: 20% (1/5) were resistant to ciprofloxacin, 20% (1/5) were resistant to cefoxitin, and 100% (5/5) were resistant to penicillin. Although these statistics suggest that antibiotic resistance was higher among strains in the CMC group, there was no significant difference between the two groups (*p* value = 0.510 using Fisher’s exact test).

Three strains of *S. aureus* with resistance to cefoxitin were identified, which characterized the strains as resistant to all beta-lactams (MRSA). Of these, two were isolated from patients whose wounds were treated with CMC hydrogel, and one was isolated from a patient treated with EGF-CMC hydrogel. Although this suggests that the incidence of MRSA was higher among strains isolated from the control group, there was no significant difference between the two groups (*p* value = 1.000 using Fisher’s exact test).

### 2.4. Microbial Load

**Figure 1:**
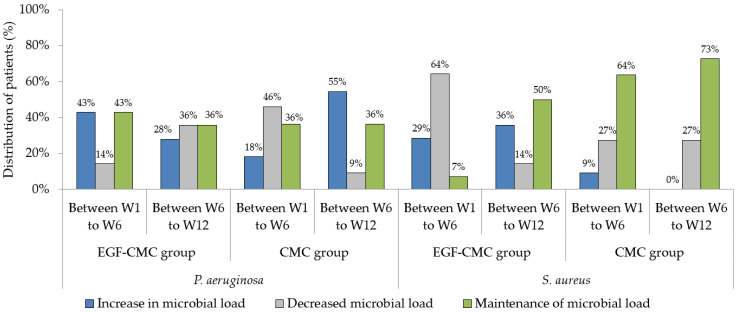
Distribution of patients according to bacterial load (using qPCR). Abbreviations: W1, Week 1; W6, Week 6; W12, Week 12.

Figure 1 shows the distribution of patients according to the bacterial loads of *S. aureus* and *P. aeruginosa* (using quantitative polymerase chain reaction (qPCR)) in the intervals from the first to the sixth week (W1 to W6) and from the sixth to the twelfth week (W6 to W12).

Concerning *P. aeruginosa*, the decreased bacterial load in patients treated with EGF was larger between W6 and W12 (36%) than between W1 and W6 (14%). On the other hand, the increased bacterial load was larger between W1 and W6 (43%) than between W6 and W12 (28%). There was no difference in the prevalence of an increased or maintained bacterial load between W1 and W6; that is, in 43% of patients, there was an increase in the load, and in another 43% there was maintenance of the bacterial load in the mentioned period. Thus, it can be said that the use of EGF in vivo primarily increased or maintained the bacterial load of *P. aeruginosa* in the first weeks of treatment.

Regarding patients treated with CMC hydrogel, the prevalence of a decreased *P. aeruginosa* load was higher between W1 and W6 (46%) than between W6 and W12 (9%). The frequency of maintenance of the *P. aeruginosa* load was 36% between W1 and W6 and between W6 and W12, demonstrating no difference between the evaluation periods. On the other hand, there was a higher frequency of an increased bacterial load between W6 and W12 (55%) than between W1 and W6 (18%), indicating that, in vivo, the microbial load of *P. aeruginosa* increased later in CMC-treated subjects.

Regarding the quantification of *S. aureus*, in patients treated with EGF-CMC there was a higher prevalence of a decreased bacterial load between W1 and W6 (64%) than between W6 and W12 (14%). An increase in the *S. aureus* load occurred more frequently between W6 and W12 (36%) than between W1 and W6 (29%). Thus, the microbial load of *S. aureus* decreased more frequently in the first weeks of treatment in patients treated with EGF-CMC hydrogel.

For patients treated with CMC hydrogel, the maintenance of the bacterial load of *S. aureus* occurred most frequently at both assessment points (64% between W1 and W6 and 73% between W6 and W12). There was a decrease in the load of *S. aureus* in 27% of patients between W1 and W6 and between W6 and W12, indicating no difference in the prevalence of a decreased *S. aureus* load between the evaluation periods. An increased bacterial load of *S. aureus* was not common between W1 and W6, as it occurred in only 9% of patients, and between W6 and W12 it did not occur in any patient.

When comparing the treatment groups, it was noted that there was a higher prevalence of a decrease in the microbial load of *P. aeruginosa* in those treated with CMC hydrogel, mainly between W1 and W6 (46%). In contrast, the decrease in the bacterial load of *S. aureus* was more prevalent in patients treated with a hydrogel containing EGF, which occurred primarily between W1 and W6 (64%).

### 2.5. Biofilm Formation Assays

All *S. aureus* isolates were biofilm producers, regardless of the EGF-CMC group. On the other hand, among *P. aeruginosa* isolates, 27.3% (3/11) of strains from the EGF-CMC group and 75% (3/4) of those isolated from the CMC hydrogel group were biofilm producers. There was no significant difference between these frequencies (*p*-value = 0.235). Overall, without differentiating species, 50% (8/16) of isolates from the EGF-CMC group and 91.7% (11/12) of isolates from the CMC group were biofilm producers, with a statistically significant difference (*p*-value = 0.039). Thus, the distribution of biofilm-producing isolates (without species distinction) was evaluated by calculating the relative risk, measured at 1.83. Therefore, the probability of isolating biofilm-producing strains from CMC-hydrogel-treated wounds was 83% greater than the probability of isolating biofilm-producing strains from EGF-CMC-treated wounds.

### 2.6. Identification of Virulence Genes

No Panton–Valentine leukocidin gene was detected in the *S. aureus* isolates, regardless of the EGF-CMC group. The *exoS* and *exoU* exoenzyme genes were not found in the *P. aeruginosa* strains identified from the CMC group. However, they were detected in four *P. aeruginosa* isolates from EGF-CMC-treated wounds (36.4%). Despite this difference between the groups, no statistical significance was observed (*p*-value = 0.077).

### 2.7. Interference of EGF in In Vitro Bacterial Growth and Biofilm Formation

**Figure 2:**
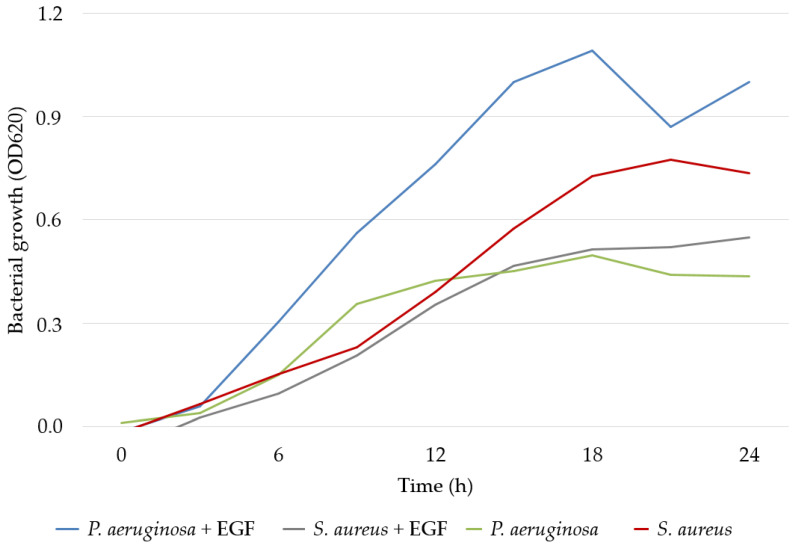
Growth of *P. aeruginosa* and *S. aureus* measured using spectrophotometry at a wavelength of 620 nm (OD620) in vitro. Abbreviations: OD620, optical density measured at 620 nanometers; EGF: epidermal growth factor.

**Figure 3:**
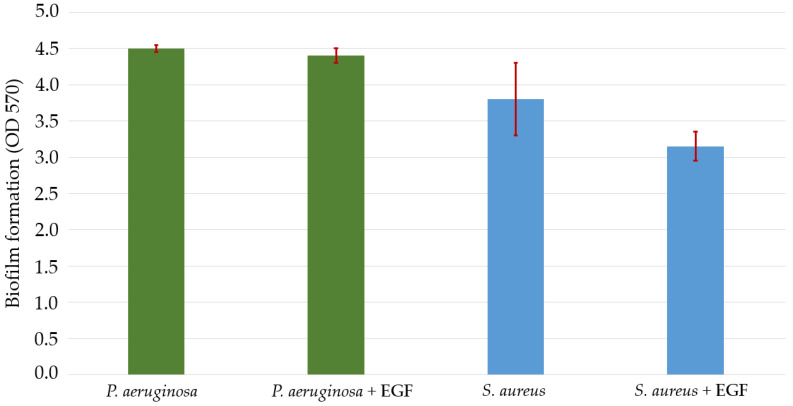
Growth of *P. aeruginosa* and *S. aureus* biofilms measured using spectrophotometry at a wavelength of 570 nm (OD570) in vitro. The error bars (highlighted in red) represent the standard deviation. Abbreviations: OD570: optical density measured at 570 nanometers; EGF: epidermal growth factor.

When evaluating the effect of EGF on the growth of *P. aeruginosa* and *S. aureus* cultures in vitro, the growth of *P. aeruginosa* was strongly stimulated and that of *S. aureus* was slightly reduced. *S. aureus* can be considered to be more impacted by EGF in terms of biofilm formation capacity since EGF caused a reduction in the expression potential of this species. In contrast, there was no change in the production of *P. aeruginosa* biofilm in the presence of EGF (Figure 2 and Figure 3).

## 3. Discussion

This research aimed to evaluate the colonization and biofilm formation of *Staphylococcus aureus* and *Pseudomonas aeruginosa* in chronic wounds of diabetic patients treated with a bioactive dressing (EGF-CMC) that consisted of a 2% carboxymethylcellulose (CMC) hydrogel loaded with epidermal growth factor (EGF). To our knowledge, this is the first study that evaluated biofilm formation in wounds treated with EGF-loaded dressings.

Regarding the health history of the patients, the results of this study were corroborated by previous studies [1,9,19] that pointed to a higher prevalence of chronic wounds in men aged 50 years or over than in younger persons.

Biofilms can represent an important virulence factor in the pathogenesis of chronic wounds, as they prolong the inflammatory phase of wound healing and consequently delay the tissue repair process [17,24].

All *S. aureus* strains were able to produce biofilms, regardless of the EGF-CMC group, which corroborated previous findings of a high prevalence of biofilm-forming strains in this species [25]. Likewise, biofilm production among *P. aeruginosa* strains is very common [24]. Our results also demonstrated that the biofilm formation capacity was significantly greater in isolates from CMC-hydrogel-treated wounds than in EGF-CMC-hydrogel-treated wounds.

A study published in 2021 [20] that evaluated biofilm formation capacity in *S. aureus* and *P. aeruginosa* strains isolated from venous ulcers treated with platelet-rich plasma (PRP) demonstrated that all strains were biofilm-forming. PRP is a platelet-rich blood derivative that is easily obtained from a patient’s blood sample after centrifugation and contains a variety of growth factors, including EGF [26].

The high incidence of isolates capable of forming biofilms and the difficulty in macroscopically visualizing these structures in wounds [17] reiterates the need to use methods that make it possible to infer the presence of biofilms in clinical practice. In vitro biofilm production assays allow the detection of bacterial adherence to an inert substrate, such as polystyrene. A high-adherence phenotype often correlates with high biofilm production capacity in vivo [27]. Thus, methods that allow the analysis of biofilm production in microorganisms isolated from chronic wounds in vitro are useful for tracking effective treatments against biofilms in vivo.

No previous study evaluated biofilm formation in microorganisms in wounds treated with EGF. Therefore, further research is suggested to elucidate the relationships between EGF and the process of bacterial biofilm formation.

Hydrogel promotes the hydration of the wound bed and, with moisture balance, provides a favorable environment for successful healing [8]. It stimulates autolytic debridement without changes in outcomes related to bacterial colonization or infection [8]. Thus, the use of hydrogel alone in the control group, compared to the results of hydrogel plus EGF, allowed an assertive assessment of the microbiological effects of EGF.

Concerning wound colonization by *P. aeruginosa*, in a prior study, the authors analyzed the effects of EGF on intralesional wound healing, focusing on risk factors for infection by this microorganism. The microorganism was found in 25% of lesions [28]. Similarly, in our study, 27.3% of wounds treated with a 2% carboxymethylcellulose gel were colonized by *P. aeruginosa*, but the percentage of wounds treated with EGF and colonized by *P. aeruginosa* was higher (57%).

Research on excisional wound healing in mice showed that a thin film of chitosan containing EGF improved wound contraction without stimulating *S. aureus* colonization [29]. Another study on mastitis treatment by *S. aureus* in sheep found that infection cure rates with an EGF treatment were similar to those obtained with the control treatment (sterile saline); thus, the authors did not recommend treating this type of infection with EGF [30].

There are reduced incidences of antimicrobial resistance in *P. aeruginosa* and *S. aureus* strains. Only one *P. aeruginosa* strain was considered multiresistant (6%), compared to three *S. aureus* strains (23%). Similar results were found in previous studies carried out in Brazil [18,19].

Molecular methods for detecting microorganisms are known to be successful, and they can be used with culture techniques to improve wound assessment. The adequate sensitivity of these methods was verified in a previous study [31].

In our research, microbial quantification showed that the use of EGF contributed to increases in or the maintenance of *P. aeruginosa* bacterial loads versus decreases in *S. aureus* levels. The analysis of the effect of EGF in vitro on microbial growth corroborated these findings. Likewise, *S. aureus* biofilm production was reduced in the presence of EGF.

In a study that evaluated *S. aureus* and *P. aeruginosa* strains in ulcers treated with PRP [20], the authors showed that bacterial load and infection presence were unrelated. On the other hand, in EGF-treated wounds, no similar studies were found, demonstrating the relevance of this investigation.

No strain of *S. aureus* with the gene encoding Panton–Valentine leucocidin was identified in previous corroborating studies [32]. This cytotoxin activates human neutrophils and is commonly found in skin and soft tissue infections [32]. A prior study analyzing strains from burns detected *exoS* and *exoU* genes in 59% and 41% of *P. aeruginosa* isolates, respectively [33]. Both were detected more frequently in a prior study compared to ours (36.4%). *ExoS* is mainly involved in bacterial colonization and invasion, and *exoU* induces cell death due to cell membrane destruction [33]. Thus, strains with these virulence factors can inhibit healing. Therefore, there is a tendency to stimulate the growth of *P. aeruginosa* in chronic wounds treated with EGF-CMC, although the same conditions seem to select strains with lower capacities for biofilm production.

This study was conducted using a convenience sample obtained in a single wound clinic in a university hospital, which is considered a limitation. Therefore, the execution of further research with larger samples is suggested because it could demonstrate other differences between the groups, with statistically significant results.

## 4. Conclusions

Most diabetic patients have chronic wounds with biofilms produced by *Staphylococcus aureus* and *Pseudomonas aeruginosa*, and successful wound healing depends on the control of these microorganisms. This randomized clinical trial aimed to evaluate the colonization and biofilm formation of *S. aureus* and *P. aeruginosa* in wounds of diabetic patients treated with a 2% carboxymethylcellulose hydrogel containing epidermal growth factor (EGF-CMC hydrogel) compared to those treated with a 2% carboxymethylcellulose hydrogel (CMC hydrogel).

EGF-CMC did not increase the bacterial growth or the microbial loads of *S. aureus* or *P. aeruginosa* compared to CMC hydrogel. Chronic wounds treated with EGF-CMC were colonized by *S. aureus* and *P. aeruginosa* strains that were less biofilm-forming than those isolated from CMC-hydrogel-treated wounds.

## 5. Materials and Methods

### 5.1. Materials

The EGF gel used in the intervention group was produced by incorporating 1 mL of a 4 ppm concentrated EGF-based oil into 150 g of a 2% carboxymethylcellulose gel. The control group was treated with a 2% carboxymethylcellulose gel without incorporating EGF (called hydrogel).

The carboxymethylcellulose gel (CMC hydrogel) was produced by the College of Pharmacy of the Fluminense Federal University (Niterói, Brazil). The amorphous gel comprised 2% carboxymethylcellulose (2 g), 0.1% methylparaben, 20% propylene glycol, and 77.9% purified water [16].

The EGF-CMC hydrogel was obtained by incorporating a commercial recombinant human epidermal growth factor (rhEFG)-based oil (EPIfactor^®®^, Infinity Pharma, Rio de Janeiro, Brazil) into CMC hydrogel in a proportion of 4000 ng/g, as described elsewhere [34]. Previous studies proved the efficiency and safety of these hydrogels for chronic wound treatment [9,16]. Both hydrogels were sterilized before the clinical assessment.

### 5.2. Study Design and Population

This randomized clinical trial was part of a larger prospective study [9] in which wounds were treated with EGF-CMC (treatment group) or CMC (control group) hydrogels. Participants were recruited based on the following inclusion criteria: an age of 18 years or older, a diagnosis of diabetes mellitus, the presence of a diabetic or venous chronic wound, an ulcer size greater than 2 cm^2^ and less than 100 cm^2^, and the presence of at least 25% granulation tissue in the wound bed. Wounds were considered chronic when they did not heal within four weeks [35]. Individuals were excluded if they had immunosuppressive diseases or clinical signs of wound infection.

This study was conducted at the Wound Repair Clinic of a university hospital in Niterói, Rio de Janeiro (Brazil). The recruitment was performed between August 2017 and December 2017. The follow-up occurred between January 2018 and July 2018. This trial was reviewed and approved by the Research Ethics Committee of the Faculty of Medicine and the Antônio Pedro University Hospital/Fluminense Federal University (Niterói, RJ, Brazil) under approval number 2,189,183 (27 July 2017). The trial was registered in the Brazilian Registry of Clinical Trials (ReBEC UTN 12616798) and followed the principles of the Declaration of Helsinki. Participants who agreed to take part signed informed consent forms.

The convenience, the sample consisted of 25 patients with diabetes mellitus who had diabetic foot ulcers or venous ulcers (14 in the EGF-CMC group and 11 in the CMC group). These 25 patients were randomly assigned to the CMC-group or the EGF-CMC group in a 1:1 ratio. The randomization code was generated by Biostat 5.0 software and was applied as the patients were enrolled. After randomization, losses during the follow-up period did not occur. The participants and statisticians were blinded to the group assignment throughout the study until the primary analysis was complete [9].

### 5.3. Study Procedures

Enrolled participants were submitted to weekly clinical assessments conducted by trained nurses during the three-month follow-up period, according to an outpatient protocol described elsewhere [9]. Wound fluid samples were collected by two research nurses using the swab culture technique described by Levine [36,37]. Biological material was collected on weeks 1 (W1), 6 (W6), and 12 (W12) and sent to a laboratory for microbial analyses.

### 5.4. Identification of S. aureus and P. aeruginosa Strains and Antimicrobial Susceptibility Tests

Swabs were added to the Stuart transport medium, placed in 2 mL of sterile saline (0.9% NaCl), and vortexed. One milliliter of this suspension was added to one milliliter of twice-concentrated trypticase soy broth (TSB) and incubated at 35 °C (±2 °C) for 24 h to 48 h.

From these cultures, plates of salted mannitol agar and cetrimide agar were inoculated and incubated at 35 °C (±2 °C) for 24 h to 48 h for the isolation of *S. aureus* and *P. aeruginosa*, respectively. Samples were identified by MALDI-TOF mass spectrometry (Microflex LT, Bruker Daltonics, Leipzig, Germany).

Antimicrobial susceptibility tests using disk diffusion were performed, and the results were analyzed according to the guidelines of the Clinical and Laboratory Standards Institute (CLSI) [38].

For *P. aeruginosa*, the following antibiotics were used: aztreonam (30 µg), ceftazidime (30 µg), ciprofloxacin (5 µg), gentamicin (10 µg), imipenem (10 µg), meropenem (10 µg), and piperacillin with tazobactam (110 µg) and polymyxin B (300 UI). The strain *P. aeruginosa* ATCC 27853 was used as a control [38].

For *S. aureus*, the following antibiotics were used: ciprofloxacin (5 μg), cefoxitin (30 μg), clindamycin (2 μg), chloramphenicol (30 μg), erythromycin (15 μg), gentamicin (10 μg), penicillin (10 UI), sulfamethoxazole+ trimethoprim (1.25 + 23.75 μg), and tetracycline (30 μg). The strain *S. aureus* ATCC 25923 was used as a test control [38].

### 5.5. Microbial Quantification Using Quantitative Real-Time Polymerase Chain Reaction (qPCR)

The quantification of *P. aeruginosa* and *S. aureus* was performed using quantitative real-time polymerase chain reaction (qPCR) with species-specific primers from DNA extracted from bacterial suspensions obtained directly from clinical wound specimens [39,40]. The DNA extraction was performed using the Wizard^®®^ Genomic DNA Purification Kit (Promega, Fitchburg, WI, USA).

Genomic DNA extracted from *P. aeruginosa* ATCC 27853 and *S. aureus* ATCC 25923 strains were used to generate a standard curve as a reference for bacterial quantification using qPCR. DNA was amplified using PCR, and amplicons were purified using an Illustra™ GFX™ kit (GE Healthcare Life Sciences, Chicago, IL, USA). The concentration of DNA was adjusted to obtain 10^5^ copies of amplicons/µL. A serial dilution was performed to obtain solutions with DNA concentrations ranging from 105 copies of amplicons/µL to 1 copy of an amplicon/µL, generating a standard curve. The primers that were used are described in Table 3.

DNA was amplified using qPCR in a standard 15 µL reaction volume, with 7.5 µL of SYBR™ Green PCR Master Mix (Applied Biosystems, Wisconsin, USA), 2 µL of DNA, and 1 µL of each primer (10 µM). Amplification was performed using a StepOne Real-Time PCR System (Applied Biosystems, Foster City, CA, USA). Standard amplification conditions were used. The dissociation curve was generated after amplification. Quantification was obtained by comparing each sample’s threshold cycle (Ct) with Cts from standard curves. The detection limit was set at 10^2^ genome copies/mL.

### 5.6. Biofilm Formation Capacity

The *S. aureus* and *P. aeruginosa* strains were inoculated in trypticase soy broth (TSB) and incubated for 24 h. Cultures were diluted 1:100 in TSB, and 100 µL of each diluted culture was transferred to a well of a 96-well polystyrene microplate (Nunclonk, Nunc, InterMed, Rochester, NY, USA) and incubated for 24 h. Later, the contents of each well were washed three times with 100 µL of phosphate-buffered saline (PBS; pH 7.4). The microplates were dried at room temperature. One hundred microliters of 0.1% crystal violet was added to each well, and the microplate was incubated for 15 min at room temperature. The dye was removed, and each well was washed three times with 100 µL of PBS. The microplates were dried at room temperature and added to 200 µL of 95% ethanol. The absorbance was measured using spectrophotometry at a wavelength of 570 nm (OD570) (UV-2600-UV-VIS spectrophotometer, SHIMADZU; Kyoto, Japan). Strains were considered biofilm producers if the OD570 values were greater than the OD570 values of the control strains [43]. *Staphylococcus epidermidis* ATCC 12228 and *P. aeruginosa* ATCC 27853 were the reference strains for positive biofilm formation.

### 5.7. Investigation of Virulence Genes

DNA from the *P. aeruginosa* and *S. aureus* strains was extracted using the Wizard^®®^ Genomic DNA Purification Kit (Promega, Fitchburg, WI, USA). Then, purified DNA was used to investigate the presence of Panton–Valentine leukocidin virulence genes (in the *S. aureus* strains) and *exo-S* and *exo-U* exoenzymes. The primers that were used are described in Table 3.

### 5.8. Interference of EGF in In Vitro Bacterial Growth and Biofilm Formation

The interference of EGF in the growth and biofilm formation of the *P. aeruginosa* ATCC 27853 and *S. aureus* ATCC 25923 strains was evaluated by measuring bacterial growth in the presence of EGF in a 96-well microplate. First, 10 µL of 0.5 standard McFarland-scale-equivalent bacterial suspensions were inoculated into wells containing 170 µL of Mueller–Hinton broth and 20 µL of EGF. The microplates were incubated for 24 h, and growth was measured every hour, using a UV-2600-UV-VIS spectrophotometer (SHIMADZU; Kyoto, Japan) at a wavelength of 620 nm (OD620) [43] to generate the growth curves.

### 5.9. Data Analysis

All variables of interest were organized in Microsoft Excel^®®^ 2007, and the data were analyzed using the Statistical Package for the Social Sciences (SPSS software, version 22.0, Armonk, NY, USA). The descriptive analysis was based on frequency and proportion distributions. In the inferential analysis, the following tests were used: the Mann–Whitney test for quantitative variables, Fisher’s exact test for categorical variables, the binomial test for differences in distribution, and relative risk (RR). All discussions of significance tests were carried out considering a maximum significance level of 5% (0.05) [44].

## Figures and Tables

**Table 1:** Initial characteristics of the wounds and the health history of the patients.

Variable	Global (*n* = 25)		EGF-CMC (*n* = 14)		CMC (*n* = 11)		*p*-Value *
	F	RF	F	RF	F	RF	
Gender							0.656 ^(a)^
Female	7	28.0%	3	21.4%	4	36.4%	
Male	18	72.0%	11	78.6%	7	63.6%	
Age (years)							0.267 ^(b)^
46|―52	1	4.0%	0	0.0%	1	9.1%	
52|―64	11	44.0%	8	57.1%	3	27.3%	
64|―76	13	52.0%	6	42.9%	7	63.6%	
ABI classification							0.536 ^(a)^
PAD mild to moderate	9	36.0%	5	35.7%	4	36.4%	
Normal	16	64.0%	9	64.3%	7	63.6%	
Glycated Hemoglobin > 7%	15	60.0%	9	64.3%	6	54.5%	0.697 ^(b)^
Injury type							1.000 ^(a)^
Diabetic	17	68.0%	9	64.3%	8	72.7%	
Venous	8	32.0%	5	35.7%	3	27.3%	
Injury area (cm^2^)							0.727 ^(b)^
2.0|―12.0	16	64.0%	8	57.1%	8	72.7%	
12.0|―|52.0	9	36.0%	6	42.8%	3	27.2%	
Exudate							0.407 ^(a)^
Serous	18	72.0%	9	64.3%	9	81.8%	
Serosanguineous	7	28.0%	5	35.7%	2	18.2%	
Exudate Amount							0.572 ^(b)^
Minimal	8	32.0%	3	21.4%	5	45.5%	
Moderate	11	44.0%	8	57.1%	3	27.3%	
Large	6	24.0%	3	21.4%	3	27.3%	
Margin							0.317 ^(b)^
Epithelized	15	60.0%	7	50.0%	8	72.7%	
Hyperkeratotic	6	24.0%	4	28.6%	2	18.2%	
Maceration	4	16.0%	3	21.4%	1	9.1%	
Granulation (% of bed that was covered)							0.851 ^(b)^
1|―50	8	32.0%	3	21.4%	5	45.5%	
51|―|100	17	68.0%	11	78.6%	6	54.6%	
Slough (% of bed that was covered)							0.317 ^(b)^
0|―25	17	68.0%	9	64.3%	8	72.7%	
26|―|100	8	32.0%	5	35.7%	3	27.3%	
Time of injury (months)							0.809 ^(b)^
Up to 6 months	4	16.0%	1	7.1%	2	18.2%	
7|―59	10	40.0%	6	42.9%	5	45.4%	
60|―|480	11	44.0%	7	50.0%	4	36.4%	

* Tests comparing variable distributions in control and intervention groups: ^(a)^ Fisher’s exact test; ^(b)^ Mann–Whitney test. Abbreviations: F: absolute frequency; RF: relative frequency; ABI: ankle–brachial index; PAD: peripheral obstructive arterial disease.

**Table 2:** Incidence of *P. aeruginosa* and *S. aureus* at three assessment points and overall incidence in the EGF-CMC and CMC groups.

Evaluation	EGF-CMC Group (*n* = 14)				CMC Group (*n* = 11)				Fisher’s Exact Test *p*-Value Comparing the Incidences in Both Groups	
	*P. aeruginosa*		*S. aureus*		*P. aeruginosa*		*S. aureus*			
	Number of Cases	Incidence	Number of Cases	Incidence	Number of Cases	Incidence	Number of Cases	Incidence	*P. aeruginosa*	*S. aureus*
Week 1	3	21.4%	3	21.4%	2	18.2%	2	18.2%	1.000	1.000
Week 6	3	21.4%	1	7.1%	1	9.1%	3	27.3%	0.604	0.288
Week 12	5	35.7%	1	7.1%	1	9.1%	3	27.3%	0.180	0.288
Just one evaluation *	8	57.1%	4	28.6%	3	27.3%	6	54.5%	0.227	0.241

* Patients who presented isolation in at least one of the three evaluations.

**Table 3:** Primers used in polymerase chain reactions.

Assay	Primer Name	5′-3′ Sequence	Size (bp)	References
Quantitative PCR of *P. aeruginosa*	PA-431-C-F	CTGGGTCGAAAGGTGGTTGTTATC	232	[39]
	PA-431-C-R	GCGGCTGGTGCGGCTGAGTC		
Virulence genes in *P. aeruginosa* strains	*exoS-F*	TCAGGTACCCGGCATTCACTACGCGG	572	[41]
	*exoS-R*	TCACTGCAGGTTCGTGACGTCTTTCTTTTA		
	*exoU-F*	CCTTAGCCATCTCAACGGTAGTC	911	[41]
	*exoU-R*	GAGGGCGAAGCTGGGGAGGTA		
Quantitative PCR of *S. aureus*	SA-442-F	TCGGTACACGATATTCTTCACA	179	[40]
	SA-442-R	ACTCTCGTATGACAGCTTC		
Virulence genes in *S. aureus* strains	lukS-PV F	GCATCAASTGTATTGGATAGCAAAAGC	463	[42]
	lukF-PV R	ATCATTAGGTAAAATGTCTGGACATGATCCA		

Abbreviations: bp, base pairs; PCR, polymerase chain reaction.

## Data Availability

The data presented in this study are available on request from the corresponding author. The data are not publicly available due to privacy.

## Abbreviation

**Abbreviation**	**Meaning**	**Page**
EGF	Epidermal growth factor	1
EGF-CMC	Epidermal growth factor loaded carboxymethylcellulose gel	1
*S. aureus*	*Staphylococcus aureus*	1
*P. aeruginosa*	*Pseudomonas aeruginosa*	1
CMC	Carboxymethylcellulose	1
ECM	Extracellular matrix	2
-CH2-COOH	Carboxymethyl group	2
FGF	Fibroblast growth factor	2
VEGF	Vascular endothelial growth factor	2
PDGF	Platelet-derived growth factor	2
EPS	Extracellular polysaccharide matrix	3
F	Absolute frequency	3
RF	Relative frequency	3
ABI	Ankle–brachial index	3
PAD	Peripheral obstructive arterial disease.	3
MRSA	Methicillin-resistant *Staphylococcus aureus*	5
qPCR	Quantitative real-time polymerase chain reaction	5
W1	First week	5
W6	Sixth week	5
W12	Twelfth week	5
*ExoS*	Exoenzyme S gene	7
*ExoU*	Exoenzyme U gene	7
OD620	Optical density measured in 620 nanometers	8
OD570	Optical density measured in 570 nanometers	8
PRP	Platelet-rich plasma	9
rhEFG	Recombinant human epidermal growth factor	11
ReBEC UTN	Brazilian Registry of Clinical Trials code	11
mL	Milliliter	11
NaCl	Sodium chloride	12
TSB	Trypticase soy broth	12
MALDI-TOF MS	Matrix-assisted laser desorption/ionization–time of flight (MALDI-TOF) mass spectrometry (MS)	12
CLSI	Clinical and Laboratory Standards Institute	12
µg	Microgram	12
ATCC	American Type Culture Collection	12
PCR	Polymerase chain reaction	12
bp	Base pairs	12
DNA	Deoxyribonucleic acid	12
Ct	Threshold cycle	13
PBS	Phosphate-buffered saline	13
SPSS	Statistical Package for the Social Sciences software	13
RR	Relative risk	14
